# Sleep disorders comorbid with ADHD: an overview of the clinical presentation and management

**DOI:** 10.1192/j.eurpsy.2024.129

**Published:** 2024-08-27

**Authors:** D. S. R. Wynchank

**Affiliations:** PsyQ, The Hague, Netherlands

## Abstract

**Abstract:**

Sleep disorders are the commonest comorbid conditions in adult ADHD. This presentation will begin with an overview of the relationship between ADHD and sleep as well as the impact of disturbed sleep on concentration, impulvity and hyperactivity. Next, we will discuss the clinical characteristics of the sleep disorders, how to screen for them and their pharmacological and non pharmacological management. By the end of the session, participants will have a clear idea of how to investigate the various sleep disorders, to distringuish and treat them.

**Disclosure of Interest:**

None Declared

